# Crockery and House-Furnishing

**Published:** 1882-04-20

**Authors:** 


CROCKERY AND HOUSE-FURNISHING.—Parties desiring anything in the house-furnishing line should visit the establishment of T. R. Ripley, of this city. Mr. Ripley is a reliable gentleman of superior business qualifications—kind, affable and courteous to visitors, and will do the straight thing by you. See his advertisement in this Journal.

THE National Board of Health *Bulletin*, of April 1st, reports cases of small-pox at the following places:—Forrest City, Ark.; Grand Rapids, Mich.; Janesville, Wis.; Lemars, Iowa; Louisville, Ky.; Moline, Ill.; Salem, Mass.; Mount Holly, Pa.; South Bethlehem, Pa.; Syracruse, N. Y.; Unionville, Ky. Only a scattering case or two in the several places mentioned, except at South Bethlehem, Pa., at which point there has been 152 cases, in a population of 5,500. Number of deaths 35. Not a single case occurred where there had been a recent vaccination.

